# The Barrier Properties of Biological Membranes Dictate How Cells Experience Oxidative Stress

**DOI:** 10.1111/mmi.15353

**Published:** 2025-03-17

**Authors:** James A. Imlay

**Affiliations:** ^1^ Department of Microbiology University of Illinois Urbana Illinois USA

**Keywords:** hydrogen peroxide, obligate anaerobiosis, oxidative stress, superoxide

## Abstract

Molecular oxygen, superoxide, and hydrogen peroxide are related oxidants that can each impair the growth of microorganisms. Strikingly, these species exhibit large differences in their abilities to cross biological membranes. This Perspective explains the basis of those differences, and it describes natural situations in which the permeability of membranes to oxidants determines the amount of stress that a bacterium experiences.

## Graphical abstract

Molecular oxygen diffuses across membranes with such speed that, despite respiration, the intracellular oxygen concentration does not differ significantly from that outside the cell. In contrast, membranes are important barriers for hydrogen peroxide and especially superoxide, so that intracellular scavenging enzymes can substantially shield cells from the effects of these external oxidants.

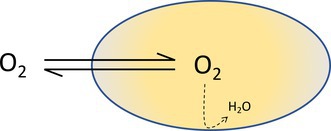

## 1 Introduction

Molecular oxygen (O_2_), superoxide (O_2_^−^), and hydrogen peroxide (H_2_O_2_) each have the capacity to disable enzymes, and when they do, they can block key metabolic pathways and suppress bacterial growth (Imlay 2013). Virtually all bacteria encounter these oxidants in some circumstances. Oxygen is present at various levels in natural environments, and even committed anaerobes confront it during transit to new habitats. Superoxide and H_2_O_2_ are routinely formed inside oxygen‐exposed cells when O_2_ adventitiously oxidizes redox enzymes, and these species can also be generated outside the cell by both biotic and abiotic processes. But to understand how these oxidants affect bacteria, it is essential to consider how easily each of these species can cross membranes. Important questions hang in the balance. Can cellular respiration shield cytoplasmic enzymes from oxygen? Will superoxide made by phagosomes penetrate into target bacteria? Does catalase protect the local community, or only those cells that contain it? Early thoughts about these questions were often incorrect, and they have been reconsidered in light of information about the barrier properties of biological membranes.

This report reviews the permeability of lipid bilayers to O_2_, O_2_^−^, and H_2_O_2_. It describes how permeability was measured and how these values were validated in living cells. It explains why this property determines how cells experience oxidative stress and how they defend themselves against it.

## 2 Molecular Oxygen

Many bacteria respire oxygen at very high rates. A well‐fed *E. coli* cell consumes 3.3 mM cytoplasmic O_2_/s—which is a lot, given that 37°C air‐saturated water contains only 0.2 mM O_2_. Effectively, the cell consumes the equivalent of its total intracellular O_2_ every 60 ms. In hypoxic environments, where the level of dissolved O_2_ might be 20‐fold lower, fewer than 5 ms would be required to exhaust the cellular oxygen content. It seems intuitively obvious, then, that respiration must lower the intracellular O_2_ concentration below what is outside the cell.

But intuition lies. Molecular oxygen is extremely small—diatomic!—and utterly nonpolar. It slides fluidly through membranes. In fact, its solubility in membranes exceeds that in water, and the experience of an O_2_ molecule as it approaches a lipid bilayer would be to suddenly whisk forward as the resistance of its solvent diminishes when O_2_ enters the membrane. Lipid bilayers are not a diffusion barrier to oxygen.

**FIGURE 1:**
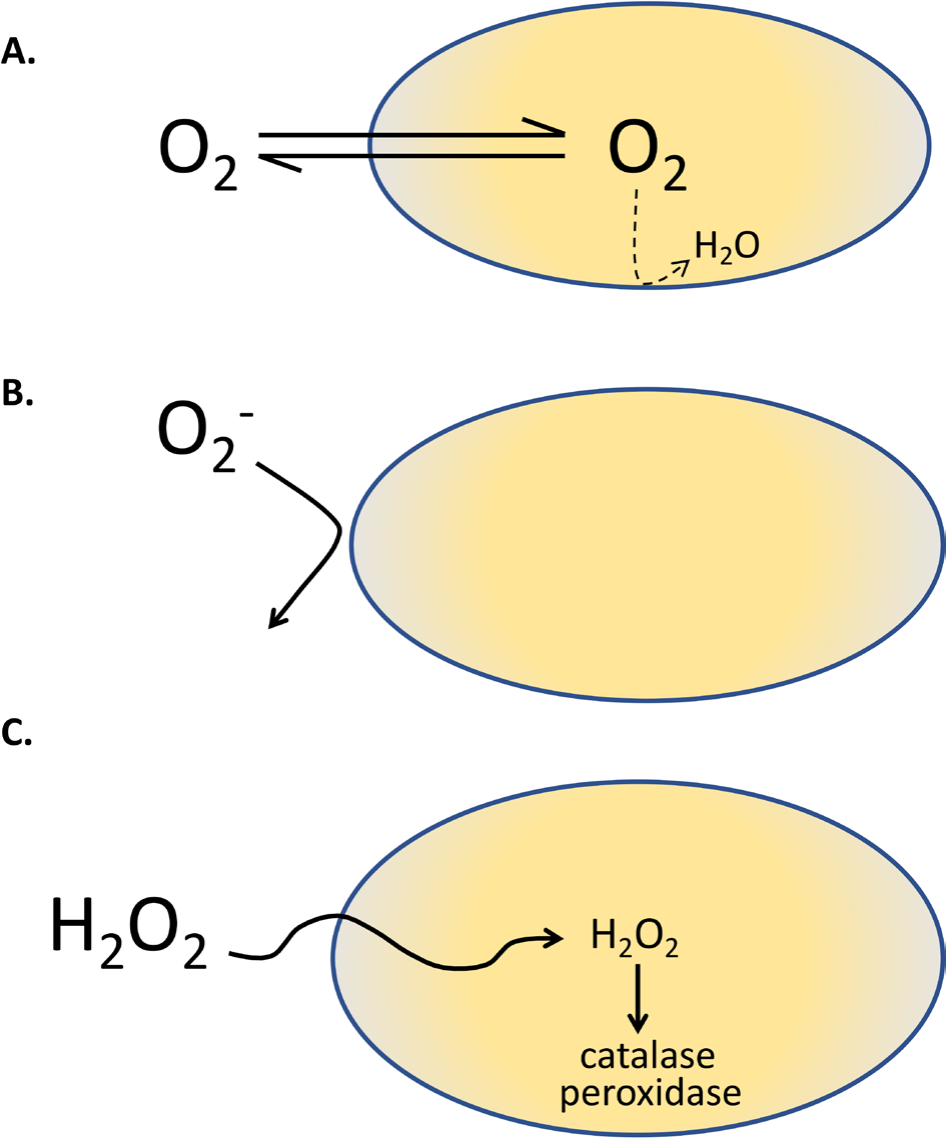
Reactive oxygen species penetrate membranes with very distinct efficiencies. (A) The high permeability coefficient of membranes for molecular oxygen (*P* = 40–120 cm‐s^−1^) enables oxygen to equilibrate across membranes far faster than it is consumed by respiring cells. Therefore, the oxygen concentration inside the cell is not significantly different than that outside the cell. (B) The anion of superoxide, which is the predominant form at physiological pH, cannot cross membranes due to its negative charge and consequent low permeability coefficient (*P* < 10^−7^ cm‐s^−1^). The conjugate acid form (HO_2_, not shown) can penetrate membranes due to its higher coefficient (*P* = 9 × 10^−4^ cm‐s^−1^) but only occurs at low pH. (C) Hydrogen peroxide (*P* = 1.6 × 10^−3^ cm‐s^−1^) crosses membranes at a moderate pace. Scavenging enzymes can lower the intracellular concentration well below that outside the cell.

Membrane permeability coefficients effectively describe the likelihood that a solute will enter and cross a membrane rather than bounce off. The membrane permeability coefficient of molecular oxygen has been determined to be 40–120 cm/s^−1^, depending upon the lipid bilayer composition and protein content (Ligeza et al. 1998; Subczynski et al. 1989; Moller et al. 2019). The low end of the range coincides with elevated protein content. This value is very high. Using it, plus measurements of oxygen consumption by well‐fed *E. coli* , one can calculate the difference in oxygen concentration between the external and internal environments (Appendix A1). The upshot is that the intracellular level is virtually equivalent to that outside the cell (Figure 1A). Molecular oxygen equilibrates across the membrane orders of magnitude more quickly than the respiratory chain consumes it.

This result has been demonstrated experimentally. Becker et al. monitored the dose of oxygen that was needed to inactivate *E. coli* Fnr, a transcription factor whose iron–sulfur cluster reacts directly with O_2_. They observed that the inactivation threshold was not lowered by mutations that abolished respiration—proving that respiration had no impact on the cytoplasmic level of oxygen (Becker et al. 1996).

In a different study, the respiration rate of intact cells was observed to be half‐maximal when external oxygen was 2 μM—the same value as the apparent K_M_ of the respiratory chain in inverted vesicles, with outward‐facing cytochrome oxidase. The implication was that even at low oxygen levels, the intracellular and extracellular concentrations are the same (Imlay and Fridovich 1991).

### 2.1 Consequences of the Free Movement of Oxygen: Tight‐Binding Cytochrome Oxidases

Due to its high membrane permeability, oxygen need not be actively transported into cells, avoiding what would otherwise be a substantial energetic cost. On the other hand, the absence of active transport means that, unlike other growth substrates, oxygen cannot be concentrated inside cells. This situation poses a particular challenge for microbes that live in low‐oxygen environments and requires that oxygen‐using enzymes evolve an extraordinarily high affinity for their substrate. *E. coli* , for example, employs as its primary respiratory ubiquinol oxidase a cytochrome *bo* enzyme with a K_M_ for oxygen of 6 μM. When oxygen levels fall lower still, the bacterium induces a *bd* oxidase with a K_M_ of 0.3 μM (Mason et al. 2009). Enzymes typically struggle to tightly bind substrates that lack shape, polarity, and charge, but they succeed with oxygen by using the half‐filled d‐orbitals of iron and copper to pair with the O_2_ di‐radical (Naqui and Chance 1986). The same tactic is also used by other oxygen‐requiring proteins, from non‐heme iron oxygenases and hemoglobins to oxygen‐sensing transcription factors like Fnr and FixL. Such high binding constants—rare among other metabolic enzymes—also enable O_2_ to outcompete competitive inhibitors like hydrogen sulfide and nitric oxide, which are commonly produced in low‐oxygen environments by the metabolic schemes of neighboring anaerobes. Consequently, one ramification of O_2_ membrane permeability is that respiring organisms depend upon access to environmental iron and copper.

### 2.2 Oxygen‐Sensitive Enzymes Require Hypoxic Habitats

Aerobic respiration, of course, is a boon to bacterial energetics. However, the dark side of oxygen is that it and its partially reduced species can damage enzymes and poison metabolism. Molecular oxygen is especially toxic to select enzymes that play critical roles in anaerobic metabolism. Because oxygen itself is a di‐radical, it directly adducts glycyl‐radical enzymes that use radical chemistry to conduct specialized reactions; pyruvate:formate lyase (PFL) and NrdD‐type ribonucleotide reductase are prominent examples (Knappe et al. 1984; Sun et al. 1996). Molecular oxygen is also a sufficiently strong univalent oxidant that it can inactivate enzymes with low‐potential redox clusters, such as pyruvate:ferredoxin oxidoreductase (PFOR) and nitrogenase. Consequently, these enzymes can function only in hypoxic or anoxic cells. Organisms that evolved to dwell in oxic environments did so by replacing PFL and PFOR with pyruvate dehydrogenase, which has no oxygen‐sensitive features, and NrdD with oxygen‐resistant ribonucleotide reductases.

The especially interesting case is that of nitrogenase, an enzyme whose function—the assimilation of nitrogen atoms from N_2_—would in principle be as valuable to oxic cells as to anoxic ones. The aerobic bacterium Anabaena solved this problem. Its secret? Every tenth or so bacterium in a cell chain is differentiated into a heterocyst, a specialized nitrogenase‐containing cell. Each heterocyst is bounded by a thickened, waxy glycolipid layer that suppresses oxygen penetration (Walsby 2007). The heterocyst receives nutrients from adjacent cells in the chain, and with minimal oxygen influx, an active cytochrome *bd* oxidase drives the O_2_ concentration to sub‐micromolar levels. These concentrations are low enough to keep nitrogenase active. Heterocysts, then, are the exception that proves the rule: normal lipid membranes are not barriers to oxygen.

Of course, the suggestion that respiration can create a protected, anoxic region is still true in the sense that the collective action of many respiring bacteria can lower the oxygen concentration within a shielded microhabitat into which oxygen has limited influx. This process enables facultative anaerobes to create local environments that permit obligate anaerobes to thrive in soil and gut (Espey 2013), and it may even be facilitated by the rubredoxin:oxygen oxidoreductases or cytochrome *bd* oxidases that many of those obligate anaerobes carry (Forte et al. 2017). These niches require a size on the scale of many microns, and they are particularly enabled by biofilms, which block convective microcurrents. Diffusion is then the sole route of oxygen entry from nearby oxic regions. Diffusion times rise in accordance with the second power of distance; therefore, whereas diffusion across a membrane (10 nm) is virtually instantaneous, penetration into a hypoxic microhabitat (> 10 μm) is extremely slow (Espey 2013).

On a practical level, experimental protocols must accommodate the fact that laboratory cultures make themselves hypoxic if the cell density is high and oxygenation is slow. This effect has been nicely quantified with *E. coli* chemostat cultures (Alexeeva et al. 2002). In the author's laboratory, the phenotypes of oxidative stress are studied in cells that are vigorously shaken in flasks filled to occupy no more than 20% of the flask volume, at cell densities no higher than 0.1 OD_600_ (ca. 3 × 10^7^ cfu/mL). Growth studies begin at 0.005 OD_600_ so that cell behavior can be observed over several generations while cultures are air‐saturated. Oxygen saturation can be achieved at higher densities if air is directly bubbled through cultures. Phenotypes on agar plates are less reliable: We suppose that the liquid layer on the surface of plates is air‐saturated, but as microcolonies grow, that local oxygen concentration is expected to diminish. Using a *lacZ* transcriptional fusion to the *tdc* operon of *Salmonella typhimurium* , Charles Miller observed that these anoxically expressed genes were strongly induced only in the center of colonies (personal communication).

### 2.3 How Did Bacteria Tolerate the Evolution of Oxygenic Photosynthesis?

The oxygen‐producing reaction center of photosystem II arose in a world that was anoxic, and it is interesting to consider why this world‐changing evolutionary step was possible. The anaerobic metabolism of that era relied upon enzymes such as PFL, PFOR, and NrdD, which are acutely oxygen‐sensitive; would not the production of oxygen immediately poison the cell that first generated it? Actually, calculations show that even if molecular oxygen were generated in that novel bacterium at the same rate as in its contemporary cyanobacterial descendants, rapid efflux across the membrane would have kept cytoplasmic oxygen at such vanishingly low concentrations [ca. 25 nM (Kihara et al. 2014)] that glycyl‐radical enzymes would have remained fully functional (Appendix A2). It was only much later, after oxygen had accumulated in the environment, that evolution was tasked with developing defensive strategies. As detailed by Mrnjavac et al. (Mrnjavac et al. 2024), the first adaptation was not the appearance of cytochrome oxidases—which would have failed to shield planktonic cells—but the evolution of oxygen‐resistant isozymes or pathways.

### 2.4 Microaerophily as a Strategy to Suppress ROS Stress

In most aerobic organisms, the primary threat of oxygen is not that it will directly damage enzymes but that it will generate O_2_^−^ and H_2_O_2_, reduced forms of oxygen that are more potent oxidants than O_2_ itself. These reactive oxygen species (ROS) are created when oxygen collides with the reduced flavins and metal centers of redox enzymes, precipitating electron transfer. Such reactions are adventitious and therefore occur in proportion to collision frequency—hence, in proportion to oxygen concentration. Most cells have evolved sufficient defenses that they can withstand the rate of ROS production that occurs in their native habitats, and they are poisoned when they are exposed to higher concentrations of oxygen. Conversely, microaerophiles are organisms that escape this threat by confining themselves to low‐oxygen niches. Interestingly, this term also fits human cells, which are exposed to lower concentrations of oxygen in situ due to the oxygen‐buffering effect of hemoglobin. Accordingly, primary cultures of mammalian cell lines often exhibit physiological defects and hypermutagenesis if they are grown outside of reduced‐oxygen incubators.

## 3 Superoxide

Lynch and Fridovich first noted that superoxide (O_2_^−^) cannot penetrate membranes (Lynch and Fridovich 1978). The pKa of the species is 4.8, which means that at a cytoplasmic pH of 7.2, the species is protonated only 0.4% of the time; the remainder of the time, its charge precludes entry into the lipid bilayer. The permeability coefficient of the protonated form—HO_2_—was evaluated by testing the ability of external superoxide to reduce cytochrome c that was enclosed inside liposomes (Korshunov and Imlay 2002). The resultant value, 0.9 × 10^−3^ cm‐s^−1^, is close to that of H_2_O (3 × 10^−3^ cm‐s^−1^ (Fettiplace 1978)), which is of similar polarity and size. Notably, these values are 10,000‐fold lower than that of O_2_. The coefficient of the O_2_^−^ anion was too low to measure (< 10^−7^ cm‐s^−1^ Figure 1B).

### 3.1 The Periplasm Is an Independent Site of Superoxide Stress

Superoxide stress then is effectively compartmentalized. Superoxide is routinely formed inside the oxic bacterial cytoplasm when oxygen oxidizes redox enzymes. This superoxide threatens other cytoplasmic enzymes that use solvent‐exposed [4Fe‐4S] clusters or Fe(II) moieties as prosthetic cofactors (Imlay 2013). Therefore, some superoxide dismutases must be located in the cytoplasm. A second group of superoxide dismutases, encoded by *sodC*, is found in the periplasms of many Gram‐negative bacteria. These enzymes scavenge superoxide that leaks from the outer face of the respiratory chain or superoxide that is made by environmental sources and that enters the periplasm through porins (Korshunov and Imlay 2006). Because superoxide cannot equilibrate across membranes, the presence of periplasmic SODs cannot compensate for the absence of cytoplasmic ones, and vice versa. Therefore, the existence of periplasmic SOD implies that there must be periplasmic or cell‐surface molecules that superoxide can attack. This is an intriguing point: The periplasm lacks iron‐cofactored enzymes of the types that superoxide is known to inactivate in the cytoplasm, meaning that a novel target of oxidation has thus far escaped detection.

*E. coli* and *Salmonella* mutants lacking periplasmic SOD do not exhibit any obvious growth defects under routine culture conditions (Gort et al. 1999; Uzzau et al. 2002). However, the virulence of several bacteria, including the model pathogen *Salmonella typhimurium* , is diminished in *sodC* mutants that lack periplasmic SOD (De‐Groote et al. 1997). *Salmonella* shares with *E. coli* a housekeeping SodCII that is primarily expressed in the stationary phase, but it also has an auxiliary SodCI that is induced during infection. Data support the inference that SodCI is specialized to defend the bacterium against superoxide that is released by the macrophage NADPH oxidase (Golubeva and Slauch 2006). This oxidase sprays superoxide into the interior of the phagosome, the compartment in which engulfed bacteria are trapped. The projected concentration of superoxide is orders of magnitude higher than in the bacterial cytoplasm (Appendix A5.1). The phagosomal program also includes a drop in pH that can approach the pKa of superoxide—which raised the possibility that protonated HO_2_ might penetrate the bacterium and disable its cytoplasmic enzymes. However, kinetic modeling suggested that the rate of entry would be moderate (Korshunov and Imlay 2002), and subsequent genetic studies confirmed that the absence of SodC did not create disabling cytoplasmic stress during infection (Craig and Slauch 2009). Therefore, it seems more likely that phagosomal HO_2_ acts by directly damaging periplasmic biomolecules (Slauch 2011). Because HO_2_ is uncharged, it should be able to abstract electrons from periplasmic biomolecules with which O_2_^−^, already an anion, cannot react. It has proven difficult to test this notion. Experimental systems have not yet produced superoxide in the quantity and duration that would replicate the dose to which bacteria are exposed in the phagosome. Consequently, the target of phagosomal superoxide remains one of the important mysteries in the fields of oxidative stress and cell‐based immunity.

### 3.2 Redox‐Cycling Drugs Are Superoxide Vectors That Circumvent the Membrane Barrier

A second route by which superoxide is weaponized is through the excretion of redox‐cycling antibiotics (Inbaraj and Chignell 2004; Turner and Messenger 1986). Walnut trees, for example, lace their leaves and seeds with juglone, a soluble quinone. When these leaves are dropped, juglone acts as an herbicide that kills undergrowth, helping seeds to find bare ground. Similarly, the lung pathogen *Pseudomonas aeruginosa* excretes pyocyanin, a redox‐active phenazine that may be involved in supporting respiration but that also can suppress the growth of competitors. In both cases, the antibiotic enters the interior of target cells and serves as a bridge that rapidly transfers electrons from redox enzymes to molecular oxygen. Internal O_2_^−^ production can rise > 20‐fold above normal levels, overwhelming defenses and inactivating metabolic enzymes (Hassan and Fridovich 1979). Bacteria that produce such antibiotics are protected by dedicated drug‐export systems (Dietrich et al. 2008), and because superoxide cannot cross membranes, the superoxide that is generated in nearby target cells will never rebound to harm the drug producer.

## 4 Hydrogen Peroxide

Hydrogen peroxide has perhaps the most interesting membrane‐permeability behavior of the reactive oxygen species—and this feature has a substantial impact upon the biology of oxidative stress. Early work tended to assume that H_2_O_2_ flows “freely” across membranes, implying that intracellular and extracellular concentrations were equilibrated, as with O_2_. Indeed, one study of *E. coli* asserted that cytoplasmic catalase did not lower H_2_O_2_ levels inside the cell below that of the surrounding environment (Ma and Eaton 1992). The authors suggested that catalase serves a communal purpose in clearing H_2_O_2_ from the local habitat rather than from the specific cell.

This inference turned out to be wrong. The membrane permeability coefficient of hydrogen peroxide was determined by measuring the rate at which intracellular scavenging enzymes can clear H_2_O_2_ from cell medium, a situation in which H_2_O_2_ entry into the cell is the rate‐limiting step (Seaver and Imlay 2001b; Winterbourn et al. 2006). The value of 1.6 × 10^−3^ cm‐s^−1^ is similar to that of H_2_O and that of protonated superoxide (HO_2_). It is orders of magnitude lower than that of molecular oxygen, in keeping with the polarity of H_2_O_2_.

At the same time, the scavenging activity of cytoplasmic peroxidases such as AhpCF was found to be quite high (Seaver and Imlay 2001b; Parsonage et al. 2005). The upshot is that scavenging activity outstrips the rate of H_2_O_2_ flow into cells, so that an outside‐to‐inside gradient arises (Figure 1C). Using data from *E. coli* , one calculates that in an environment containing micromolar H_2_O_2_, the steady‐state H_2_O_2_ concentration in the cytoplasm may be 10‐fold lower (Imlay 2013) (Appendix A3). This effect was overlooked in the earlier studies because they had employed unnaturally high (millimolar) concentrations of H_2_O_2_ that inactivated the peroxidase system.

This conclusion has been verified in several ways. Notably, a scavenging‐proficient cell fails to protect a peroxidase/catalase mutant when they are cocultured (Seaver and Imlay 2001b). Were H_2_O_2_ to equilibrate between intracellular and extracellular environments, both cells would experience the same degree of internal H_2_O_2_ stress.

### 4.1 Inducible Scavenging Systems Effectively Shield the Cell Interior From Environmental H_2_O_2_

Extracellular hydrogen peroxide is generated in microbial habitats by a number of processes: photochemistry, chemical reactions at oxic:anoxic interfaces, the metabolism of lactic acid bacteria, and the oxidative burst of phagocytes (Imlay 2018). Most microbes possess transcription factors in their cytoplasms, such as OxyR and PerR, whose role is to detect and respond to this H_2_O_2_ when it flows into the cell (Zheng et al. 1998; Sen and Imlay 2021). Experiments with *E. coli* indicate that 100–200 nM intracellular H_2_O_2_ is sufficient to activate its OxyR system (Aslund et al. 1999; Seaver and Imlay 2001b). This level of cytoplasmic H_2_O_2_ is reached when 3 micromolar H_2_O_2_ is provided in the growth medium (Li and Imlay 2018)—which is consistent with the idea that the combination of high scavenger activity and sluggish H_2_O_2_ movement across the membrane creates a > 10‐fold gradient of H_2_O_2_. The 3 micromolar value is near the upper range of H_2_O_2_ levels that have been observed in such diverse environments as streams, open waters, and the bloodstream (Forman et al. 2016; Morris et al. 2022). Microfluidic studies have shown that similar concentrations elicit stress responses in other bacteria as well (Padron et al. 2023).

Single‐cell experiments with microfluidics have nicely demonstrated that induced scavenging enzymes generate a strong transmembrane concentration gradient. When *E. coli* was exposed to a constant flow of 100 micromolar H_2_O_2_, genes controlled by OxyR were quickly and strongly induced—and then their expression subsided to a lower constant level (Lagage et al. 2022). As intracellular scavenging enzymes became abundant, the activity of OxyR diminished, despite the persistence of the extracellular dose.

Among the genes that are induced by OxyR are the scavenging enzymes AhpCF and catalase, as well as enzymes that protect iron and DNA (Sen and Imlay 2021). As AhpCF and catalase titers rise, the outside‐to‐inside gradient will become even steeper. Indeed, even though 0.3 μM intracellular H_2_O_2_ can be enough to poison biosynthetic pathways (Sobota et al. 2014), *E. coli* can continue to grow in minimal medium when the extracellular H_2_O_2_ rises as high as 10 micromolar (Li and Imlay 2018). In bacteria with higher activities of scavenging enzymes, the gradient—and tolerated levels of environmental H_2_O_2_—may be even greater.

### 4.2 An Extracytoplasmic Peroxidase Exploits the Transmembrane Gradient

This startling tolerance of the bacterium for H_2_O_2_ is underscored by the fact that OxyR even induces a cytochrome c peroxidase (Ccp) (Khademian and Imlay 2017) that exploits H_2_O_2_ as a growth substrate. Ccp is a membrane‐bound protein that catalyzes electron transfer from reduced respiratory quinones to H_2_O_2_, and it thereby allows the cell to employ H_2_O_2_ as a terminal electron acceptor for respiration. The Ccp active site is located on the periplasmic face of the membrane; this arrangement allows the internal scavenging enzymes to keep cytoplasmic H_2_O_2_ below bacteriostatic levels, while Ccp still has access to the higher H_2_O_2_ concentration in the periplasm. The K_M_ for H_2_O_2_ of Ccp—5 micromolar—nicely fits the environmental H_2_O_2_ concentration that is needed to activate its inducer. Therefore, the barrier property of the membrane allows *E. coli* not only to tolerate H_2_O_2_ but also to thrive in it.

### 4.3 Phagosome‐Generated H_2_O_2_ Is Unlikely to Kill Captive Bacteria

From this perspective, it is instructive to consider whether a bacterium is affected by the H_2_O_2_ that is formed in phagosomes during the oxidative burst. The dismutation of superoxide generates the H_2_O_2_. In macrophages, the H_2_O_2_ will rise to a steady‐state level that is limited by the fact that the H_2_O_2_ will steadily flow across membranes—not only into the captive bacterium but also out of the phagosome. In neutrophils, H_2_O_2_ production is faster, but the H_2_O_2_ is simultaneously consumed by myeloperoxidase. Labs have projected that the H_2_O_2_ levels rise no higher than 1–4 micromolar in macrophage phagosomes (Imlay 2009; Appendix A5.2) and 2 micromolar in neutrophil phagosomes (Winterbourn et al. 2006), which are well within the range that *E. coli* can tolerate. Higher concentrations would be achieved only if H_2_O_2_ were to accumulate in the regional tissue, as might be the case during chronic inflammation—a situation that would be suppressed if host cells scavenged the local H_2_O_2_. Hydrogen peroxide itself, then, seems unlikely to be a main bacteriocide in phagocytes. Its precursor superoxide and downstream product hypochlorous acid in neutrophils (Sultana and Dahl 2023) seem more likely to be the agents that kill bacteria.

### 4.4 In Laboratory Cultures H_2_O_2_ Stress Primarily Stems From Media

Aerobic cells continuously generate internal H_2_O_2_ at a substantial pace: ca. 15 μM/s in the model organism *E. coli* (Seaver and Imlay 2001b). Against this backdrop, one might wonder whether H_2_O_2_ influx from the environment comprises a significant additional threat. In fact, calculations (Appendix A4) show that 0.2 μM external H_2_O_2_ creates an influx equivalent to the pace of internal H_2_O_2_ production. Therefore, if cells enter an environment containing, say, 2 μM H_2_O_2_, the total H_2_O_2_ within the cell interior immediately rises tenfold. This situation is very pertinent to laboratory experiments because most sterile media contain at least this much H_2_O_2_, which is generated either by photochemistry under lab lights or by the chemical oxidation of sugars (Li and Imlay 2018). The stress is substantial enough that isolated colonies of *E. coli oxyR* mutants cannot form when the cells are deposited on standard LB or glucose plates (Ezraty et al. 2014; Li and Imlay 2018). It turns out that every time workers streak wild‐type *E. coli* upon plates, the cells survive only because they induce this stress response. A similar effect has been documented when sterile LB is delivered to *Pseudomonas aeruginosa* in microfluidic chambers (Padron et al. 2023). In fact, the inability of some environmental bacteria to grow in lab media apparently derives from their inability to contend with the H_2_O_2_ that those media contain (Martin et al. 1976; Tanaka et al. 2014; Dione et al. 2016).

## 5 Hydroxyl Radical

I close this article with the hydroxyl radical. It warrants mention because it is the most powerful of reactive oxygen species, reacting readily with virtually all biomolecules (Davies 2005). It is the oxygen species that can oxidize DNA and potentially kill cells, rather than merely inhibit their growth. Hydroxyl radicals are formed in biological systems by the Fenton reaction, in which cytoplasmic Fe(II) atoms transfer an electron to H_2_O_2_. The hydroxyl radical is tiny and uncharged, and in principle, it might be expected to cross membranes relatively freely. However, its reactivity—with nucleic acids, proteins, lipids, or anything else—is so great that it is believed to oxidize nearby biomolecules almost immediately at its site of formation (Chevion 1988; Imlay et al. 1988). Therefore, the destructive effects of hydroxyl radicals are focused upon iron‐rich compartments, and in particular upon biomolecules that bind iron. In most natural habitats, the environmental concentration of loose iron is low (Wandersman and Delepelaire 2004), and it seems doubtful that cell‐surface oxidation by hydroxyl radicals is a common event.

## 6 Conclusion

The membrane permeability of reactive oxygen species has a large imprint upon how cells cope with oxidative environments. Molecular oxygen equilibrates across membranes, superoxide barely crosses them at all, and hydrogen peroxide is somewhere in the middle. These distinctions have guided how cells defend themselves against them. Respiration does not make a cell hypoxic relative to its immediate environment, and so oxygen‐sensitive cells must protect themselves by seeking hypoxic habitats. Superoxide is an especially toxic compound that does not cross membranes, and so cells need to implant a superoxide dismutase or reductase in each cellular compartment in which it is formed. Hydrogen peroxide crosses membranes at a moderate pace. Cells can therefore be threatened by external H_2_O_2_—but they also can defend themselves by degrading it as quickly as it enters.

## Author Contributions

**James A. Imlay:** conceptualization, formal analysis, writing – original draft, writing – review and editing.

## Data Availability

Data sharing is not applicable to this article as no new data were created or analyzed in this study.

## Appendix A

### Calculations Underpinning the Main Text

#### A1. What Is the O_2_ Concentration Inside Well‐Fed *E. coli* Growing in Air‐Saturated Medium?

1. At 37°C, the concentration of oxygen in air‐saturated medium is 210 μM.
2. This calculation uses 40 cm–s^−1^ as a low limit for the membrane permeability coefficient *P*. (Ligeza et al. 1998)
3. In LB medium, the surface area (SA) of *E. coli* is 1.4 × 10^−7^ cm^2^‐cell. (Seaver and Imlay 2001b)
4. The rate of oxygen consumption in LB medium is 1.23 × 10^−17^ mol‐cell^−1^. (Imlay and Fridovich 1991). The respiration rate under this rich growth condition is likely to be near‐maximal for *E. coli* .

The rate of oxygen entry into the cell equals the sum of the rate of oxygen exit plus oxygen consumption by respiration:

1. *V*_entry_ = *V*_exit_ + *V*_respiration_.

The rate of flow across the membrane (in either direction) = [O_2_, M] × P × SA × (10^−3^ L‐cm^3^).

Then:

2 *V*_entry_ = (210 × 10^−6^ mol/L) × (40 cm‐s^−1^) × (1.4 × 10^−7^ cm^2^‐cell^−1^) × (10^−3^ L‐cm^3^).
3 *V*_exit_ = [O_2_,_in_, mol/L] × (40 cm‐s^−1^) × (1.4 × 10^−7^ cm^2^‐cell^−1^) × (10^−3^ L‐cm^−3^).
4 *V*_respiration_ = 1.23 × 10^−17^mol‐cell^−1^.

By substituting 2–4 into equation 1, one calculates that the O_2_ concentration inside the cell is 209.998 μM, which is only 0.001% lower than the external O_2_ concentration.

Even if external oxygen were far lower—5 μM, which approaches the K_M_ of the respiratory chain—then a similar calculation shows that the O_2_ concentration inside the cell is 4.998 μM, which is 0.04% lower than the external O_2_ concentration. (At even lower O_2_ concentrations respiration will slow, suppressing the formation of an oxygen gradient.)

In conclusion, oxygen flow across membranes is far faster than respiration; consequently, the cell interior remains as oxic as the immediate environment.

#### A2. Would Intracellular O_2_ Production Inactivate Glycyl‐Radical Enzymes in an Ancient Microbe Performing Oxygenic Photosynthesis?

1. In 0°C water equilibrated with 100% oxygen, the concentration of dissolved oxygen is approximately 2.19 mM (Xing et al. 2014).
2. In RT water equilibrated with air, the dissolved oxygen is approximately 280 μM.
3. Measurements showed the half‐life of pyruvate:formate lyase was 10 s in 0°C water equilibrated with 100% oxygen.
4. Modeling indicates 25 nM O_2_ in a cyanobacterium photosynthesizing at a contemporary rate in an anoxic environment.

The O_2_ level in a cyanobacterium producing O_2_ at contemporary rates in an anoxic environment was calculated by Kihara et al. to be 25 nM (Kihara et al. 2014). This level reflects O_2_ diffusing out of a cyanobacterium of standard size into an external habitat devoid of oxygen, so that no re‐entry occurs. The O_2_ level is determined by the time needed for O_2_ to diffuse away from that point source in water; the membrane itself does not comprise a barrier, and the O_2_ level would be very slightly higher without it (as O_2_ diffuses slightly more slowly through a layer of water than a layer of membrane).

The rate constant for inactivation of the enzyme can be calculated using the data above:

$$\mathrm{Ln}\;\left(N/\mathrm{No}\right)\;=\;-k\;\left[O_{2}\right]\;t$$

for a reaction that is first‐order in oxygen concentration. The 10‐s half‐life determined in buffer saturated with 100% O_2_ (Knappe et al. 1984) indicates that *k* = 31.7 M^−1^ s^−1^. The oxygen concentration in air‐equilibrated RT water is 280 μM, so the half‐life would be 77 s. But at 25 nM, the half‐life would be 10 days.

This calculation presumes that temperature does not directly affect the rate constant—that is, that the reaction has a minimal energy of activation. This assumption is likely to be approximately true for a radical‐radical reaction such as that between a glycyl radical and molecular oxygen. (Even a conventional estimate of a doubling of rate constant per 10° would shorten the half‐life at RT to no fewer than 2 days.)

We conclude that endogenous O_2_ production would not have elevated O_2_ concentration in an ancient bacterium to a level sufficient to poison its glycyl‐radical enzymes. Over time, however, O_2_ gradually accumulated in the environment as reducing species were progressively titrated. This environmental change imposed selective pressure for the acquisition of oxygen‐resistant enzymology. Adaptations included the replacement of glycyl‐radical ribonucleotide reductases and pyruvate:formate lyase with the oxygen‐tolerant ribonucleotide reductases and pyruvate dehydrogenase that are found in contemporary cyanobacteria.

#### A3. Does the Semi‐Permeable Nature of Membranes Allow Intracellular Scavenging Enzymes to Protect an Isolated Cell From H_2_O_2_?

1. In LB medium, the surface area (SA) of *E. coli* is 1.4 × 10^−7^ cm^2^‐cell (Seaver and Imlay 2001b).
2. This calculation uses 1.6 × 10^−3^ cm‐s^−1^ as the membrane permeability coefficient P of H_2_O_2_ (Seaver and Imlay 2001b).
3. The turnover of uninduced catalase inside an *E. coli* cell was determined to fit the equation: rate of catalase = [H_2_O_2_ in, M] × 2.7 × 10^−13^ L/s (Seaver and Imlay 2001b).
4. The turnover of uninduced AhpCF inside an *E. coli* cell was determined to fit the Michaelis–Menten‐based equation: rate of AhpCF = [H_2_O_2_ in, M] × {(2.1 × 10^−18^ mol/s)/([H_2_O_2_ in, M] + 1.2 × 10^−6^ M)} (Seaver and Imlay 2001b).

$$\begin{array}{c} V_{\text{entry}}=\left[H_{2}O_{2\text{out}}\right]\times P\times \mathrm{SA}\times 10^{-3}\;L/{\mathrm{cm}}^{3}\\ =\left[H_{2}O_{2\text{out}}\right]\times \left(1.6\times 10^{-3}\;\mathrm{cm}-s^{-1}\right)\;x\;\left(1.4\times 10^{-7}\;{\mathrm{cm}}^{2}-\text{cell}\right)\times 10^{-3}\;L/{\mathrm{cm}}^{3} \end{array}$$

$$V_{\text{exit}}=\left[H_{2}O_{2\text{in}}\right]\times \left(1.6\times 10^{-3}\;\mathrm{cm}-s^{-1}\right)\times \left(1.4\times 10^{-7}{\mathrm{cm}}^{2}-\text{cell}\right)\times 10^{-3}\;L/{\mathrm{cm}}^{3}$$

$$V_{\text{catalase}}\;=\;\left[H_{2}O_{2\text{in}},\;M\right]\times 2.7\times 10^{-13}\;L/s$$

$$V_{\text{AhpCF}}\;=\left[H_{2}O_{2\text{in}},\;M\right]\times \left\{\left(2.1\times 10^{-18}\;\mathrm{mol}/s\right)/\left(\left[H_{2}O_{2\text{in}},\;M\right]+1.2\times 10^{-6}\;M\right)\right\}.$$

When H_2_O_2out_ = 5 μM, the solution to this equation is that [H_2_O_2 in_] = 0.7 μM. That is, the intracellular H_2_O_2_ concentration is 1/7th the external concentration. Under these conditions, the fate of intracellular H_2_O_2_ is that 69% would be scavenged by AhpCF, 17% would be scavenged by catalase, and 14% would efflux from the cell. At lower concentrations of extracellular H_2_O_2_, the out‐to‐in gradient approaches a limit of 10‐fold, with 78% of internal H_2_O_2_ scavenged by AhpCF, 12% by catalase, and 10% lost by efflux.

Notably, the AhpCF activity measurements used for this calculation represented a lower limit for its activity. Measurements of AhpC content and turnover number (Link et al. 1997; Parsonage et al. 2005) suggest a modestly higher rate (Imlay 2013)—which would indicate the gradient is steeper than calculated here.

At high concentrations of H_2_O_2_, AhpCF is saturated (and perhaps inactivated). Under those conditions, the gradient is diminished. The equations suggest that at 100 μM external H_2_O_2_, the intracellular H_2_O_2_ would be 41 μM, only a 2.4‐fold gradient. At 1 mM external H_2_O_2_, the intracellular H_2_O_2_ would be 450 μM, representing a 2.2‐fold gradient; 45% flows back out of the cell, 54% is scavenged by catalase, and only 1% is scavenged by AhpCF. The absence of the gradient at high H_2_O_2_ concentrations is the reason that workers concluded that the benefit of scavenging was to rid the environment, rather than the individual cell, of H_2_O_2_. Those concentrations are non‐physiological (Imlay 2018).

At all concentrations, the gradient will grow when AhpCF and catalase are induced by OxyR.

#### A4. How Much Environmental H_2_O_2_ Is Needed to Make Exogenous H_2_O_2_ a Greater Stress Upon the Cell Interior Than Endogenous H_2_O_2_?

Hydrogen peroxide and O_2_^−^ are continually produced inside aerobic cells due to the adventitious oxidation of redox enzymes. The rate of H_2_O_2_ production inside well‐fed, fully aerated *E. coli* has been directly measured using strains that lack scavenging enzymes: 15 μM/s (Seaver and Imlay 2001a). For a typical cell of volume 3.2 × 10^−15^ L (Imlay and Fridovich 1991), this rate represents 5 × 10^−20^ mol/s‐cell. The basal activity of AhpCF has been estimated to keep the steady‐state H_2_O_2_ concentration at approximately 50 nM (Imlay 2013). Given this high rate of internal production, how much H_2_O_2_ must be in the external medium for the influx from the environment to exceed the rate of internal production?

From the previous section:

Setting this rate equal to 5 × 10^−20^ mol/s, one calculates that the entry rate is 15 μM/s (5 × 10^−20^ mol/s‐cell) when the external H_2_O_2_ concentration is 0.2 μM. At higher levels, most of the H_2_O_2_ experienced by the cell cytoplasm flows in from the environment rather than originating from internal sources. This is notable, as in many environmental fluids—and most growth media—H_2_O_2_ levels may be substantially higher.

In contrast, because O_2_^−^ does not cross membranes, intracellular O_2_^−^ stress arises exclusively from O_2_^−^ that is made internally.

#### A5. What Are the Concentrations of Superoxide and Hydrogen Peroxide Inside Macrophages?

Actual measurements of these concentrations are not available, but estimates can be made based upon the rate of superoxide formation, the approximate dimensions of the phagosome, rate constants for superoxide dismutation, and the membrane permeability coefficient of H_2_O_2_ (Imlay 2009).

##### A5.1 Superoxide

Under steady‐state conditions, the rate of superoxide formation equals its rate of dismutation. In the phagosomes of neutrophils, superoxide formation has been measured to occur at several millimolar per second (Jiang et al. 1997; Winterbourn et al. 2006), while the rate is an order of magnitude lower in the phagosomes of macrophages. Using 0.5 mM/s as an estimate and recognizing that spontaneous superoxide dismutation occurs by comproportionation of protonated and deprotonated superoxide, the steady‐state equation is established:

$$0.5\times 10^{-3}\;M/s\;=k\;\times \;\left[{O_{2}}^{-}\right]\times \left[{\mathrm{HO}}_{2}\right]$$

The value of *k* is 8 × 10^7^ M^−1^ s^−1^ and the pKa of superoxide is 4.8 (Fridovich 1978), so at pH 7.4, [HO_2_] = 2.5 × 10^−3^ × [O_2_^−^]. Therefore,

$$0.5\times 10^{-3}\;M/s=\left(8\times \;10^{7}\;M^{-1}\;s^{-1}\right)\times \left(2.5\times 10^{-3}\right)\times {\left[{O_{2}}^{-}\right]}^{2}$$

And [O_2_^−^] = 50 μM. The concentration of [HO_2_] = 0.1 μM.

(The half‐life of O_2_^−^ under these conditions is 70 ms, justifying the use of steady‐state kinetics to describe an oxidative burst that lasts minutes to hours).

This calculated 50 μM superoxide in the phagosome is very high, exceeding by four orders of magnitude the 0.2 nM concentration of superoxide that has been predicted to obtain inside the *E. coli* cytoplasm (Imlay 2013).

Because the pH of phagosomes can drop below 5, it is worth predicting the superoxide concentrations under this condition. Using the approach above, one calculates that at pH 6 the steady‐state [O_2_^−^] = 10 μM and [HO_2_] = 0.6 μM. At pH 4.5, [O_2_^−^] = 2 μM and [HO_2_] = 4 μM. This high concentration of HO_2_. raises the possibility that this species, rather than the O_2_^−^ anion, mediates the toxic action of phagosomal NADPH oxidase. Because HO_2_ is uncharged, this situation can also drive into the bacterial cytoplasm an influx of superoxide that moderately exceeds the rate of endogenous superoxide formation (Imlay 2013).

These calculations depend upon measurements of superoxide production that have not been precisely measured and that may vary with conditions. However, because the rate of superoxide dismutation is second‐order in superoxide concentration, the steady‐state concentrations that were calculated here vary as the square root of the superoxide flux—that is, if superoxide production were doubled, the steady‐state concentrations would rise by only 40%. Thus, the conclusions are relatively robust.

##### A5.2. Hydrogen Peroxide

If the rate of superoxide production in a phagosome were 0.5 mM/s (above), then due to dismutation, the rate of H_2_O_2_ production would be 0.25 mM/s. The H_2_O_2_ will be cleared from the phagosome by diffusion across membranes either into the captive bacterium or out of the phagosome. Microscopy suggests that the phagosomal volume is approximately twice that of the bacterium. If the bacterium were modeled as a cylinder of length 3.7 μm and radius 0.53 μm (Korshunov and Imlay 2002), its surface area per cell would be 1.2 × 10^−7^ cm^2^ and its volume would be 3.3 × 10^−15^ L. The phagosomal membrane would have a length of 4.7 μm and radius 0.67 μm, and its surface area would be 2.0 × 10^−7^ cm^2^. The rate of H_2_O_2_ production can then be equated to the rate of its egress across a total membrane surface area (SA) of 3.2 × 10^−7^ cm^2^ (Imlay 2009). Using the interstitial volume as 3.3 × 10^−15^ L and denoting the permeability coefficient as *P*:

$$H_{2}O_{2}\;\text{formation}=\left[H_{2}O_{2}\right]\times 10^{-3}\;L-{\mathrm{cm}}^{-3}\times P\;\left(\mathrm{cm}/s\right)\times \mathrm{SA}\;\left({\mathrm{cm}}^{2}\right)$$

$$\begin{array}{cc} \left(0.25\times 10^{-3}M/s\right)\times \left(3.3\times 10^{-15}L\right)= & \left[H_{2}O_{2}\right]\times \left(10^{-3}L/{\mathrm{cm}}^{3}\right)\times \left(1.6\times 10^{-3}\mathrm{cm}/s\right)\\ & \times \left(3.2\times 10^{-7}{\mathrm{cm}}^{2}\right) \end{array}$$

The steady‐state H_2_O_2_ concentration would be 1.6 μM.

This calculation relies upon approximations, but credible adjustments—for a different cell size, a different ratio of phagosome‐to‐cell volume, or membrane invaginations—would not substantially change the prediction that phagosomal H_2_O_2_ levels are in the low‐micromolar range. Such concentrations approach the level sufficient to induce OxyR in wild‐type *E. coli* , but they fail to block its growth even in minimal medium (Imlay 2009).

Interestingly, although neutrophils produce H_2_O_2_ much more rapidly than do macrophages, the H_2_O_2_ is simultaneously consumed by myeloperoxidase. Winterbourn and colleagues have calculated that the steady‐state H_2_O_2_ concentration in neutrophil phagosomes is similar to that within macrophage phagosomes (Winterbourn et al. 2006).
